# Some New Hermite–Hadamard and Related Inequalities for Convex Functions via (*p*,*q*)-Integral

**DOI:** 10.3390/e23070828

**Published:** 2021-06-29

**Authors:** Miguel Vivas-Cortez, Muhammad Aamir Ali, Hüseyin Budak, Humaira Kalsoom, Praveen Agarwal

**Affiliations:** 1 Faculty of Exact and Natural Sciences, School of Physical Sciences and Mathematics, University of Buenos Aires, Av. 12 de octubre 1076 y Roca, Apartado Postal 17-01-2184, Sede Quito, Ecuador; mjvivas@puce.edu.ec; 2 Jiangsu Key Laboratory for NSLSCS, School of Mathematical Sciences, Nanjing Normal University, Nanjing 210023, China; 3 Department of Mathematics, Faculty of Science and Arts, Düzce University, Düzce 81620, Turkey; hsyn.budak@gmail.com; 4 Department of Mathematics, Zhejiang Normal University, Jinhua 321004, China; 5 Department of Mathematics, Anand International College of Engineering, Jaipur 303012, India; goyal.praveen2011@gmail.com; 6 Nonlinear Dynamics Research Center (NDRC), Ajman University, Ajman 346, United Arab Emirates; 7 International Center for Basic and Applied Sciences, Jaipur 302029, India

**Keywords:** quantum calculus, post-quantum calculus, (*p*,*q*) estimates for midpoint and trapezoidal type inequalities

## Abstract

In this investigation, for convex functions, some new (p,q)–Hermite–Hadamard-type inequalities using the notions of ${\left(p,q\right)}^{\pi_{2}}$ derivative and ${\left(p,q\right)}^{\pi_{2}}$ integral are obtained. Furthermore, for ${\left(p,q\right)}^{\pi_{2}}$-differentiable convex functions, some new (p,q) estimates for midpoint and trapezoidal-type inequalities using the notions of ${\left(p,q\right)}^{\pi_{2}}$ integral are offered. It is also shown that the newly proved results for p=1 and $q\to 1^{-}$ can be converted into some existing results. Finally, we discuss how the special means can be used to address newly discovered inequalities.

## 1. Introduction

In convex functions theory, Hermite–Hadamard (HH) inequality, which was discovered by C. Hermite and J. Hadamard independently, is very important (see also [1,2] (p. 137)):

(1) $$\Pi \left(\frac{\pi_{1}+\pi_{2}}{2}\right)\leq \frac{1}{\pi_{2}-\pi_{1}}\int_{\pi_{1}}^{\pi_{2}}\Pi \left(x\right)dx\leq \frac{\Pi \left(\pi_{1}\right)+\Pi \left(\pi_{2}\right)}{2}$$

where Π is a convex function. In the case of concave mappings, the above inequality is satisfied in reverse order.

On the other hand, in the domain of *q* analysis, many works are being carried out as initiated by Euler in order to attain adeptness in mathematics that constructs quantum computing *q* calculus considered as a relationship between physics and mathematics. In different areas of mathematics, it has numerous applications such as combinatorics, number theory, basic hypergeometric functions, orthogonal polynomials, and other sciences, as well as mechanics, the theory of relativity, and quantum theory [3,4]. Quantum calculus also has many applications in quantum information theory, which is an interdisciplinary area that encompasses computer science, information theory, philosophy, and cryptography, among other areas [5,6]. Apparently, Euler invented this important branch of mathematics. He used the *q* parameter in Newton’s work on infinite series. Later, in a methodical manner, the *q*-calculus, calculus without limits, was firstly given by Jackson [7,8]. In 1966, Al-Salam [9] introduced a *q*-analogue of the *q*-fractional integral and *q*-Riemann–Liouville fractional. Since then, related research has gradually increased. In particular, in 2013, Tariboon introduced the ${}_{\pi_{1}}D_{q}$-difference operator and $q_{\pi_{1}}$-integral in [10]. In 2020, Bermudo et al. introduced the notion of ${}^{\pi_{2}}D_{q}$ derivative and $q^{\pi_{2}}$-integral in [11]. Sadjang generalized to quantum calculus and introduced the notions of post-quantum calculus, or briefly $\left(p,q\right)$-calculus in [12]. In [13], Tunç and Göv gave the post-quantum variant of ${}_{\pi_{1}}D_{q}$-difference operator and $q_{\pi_{1}}$-integral. Recently, in 2021, Chu et al. introduced the notions of ${}^{\pi_{2}}D_{p,q}$ derivative and ${\left(p,q\right)}^{\pi_{2}}$-integral in [14].

Many integral inequalities have been studied using quantum and post-quantum integrals for various types of functions. For example, in [11,15,16,17,18,19,20,21,22,23,24,25], the authors used ${}_{\pi_{1}}D_{q}{,}^{\pi_{2}}D_{q}$-derivatives and $q_{\pi_{1}},q^{\pi_{2}}$-integrals to prove Hermite–Hadamard integral inequalities and their left–right estimates for convex and coordinated convex functions. In [26], Noor et al. presented a generalized version of quantum integral inequalities. For generalized quasi-convex functions, Nwaeze et al. proved certain parameterized quantum integral inequalities in [27]. Khan et al. proved quantum Hermite–Hadamard inequality using the green function in [28]. Budak et al. [29], Ali et al. [30,31], and Vivas-Cortez et al. [32] developed new quantum Simpson’s and quantum Newton’s type inequalities for convex and coordinated convex functions. For quantum Ostrowski’s inequalities for convex and co-ordinated convex functions, one can consult [33,34,35]. Kunt et al. [36] generalized the results of [18] and proved Hermite–Hadamard-type inequalities and their left estimates using ${}_{\pi_{1}}D_{p,q}$ difference operator and ${\left(p,q\right)}_{\pi_{1}}$ integral. Recently, Latif et al. [37] found the right estimates of Hermite–Hadamard type inequalities proved by Kunt et al. [36]. To prove Ostrowski’s inequalities, Chu et al. [14] used the concepts of ${}^{\pi_{2}}D_{p,q}$ difference operator and ${\left(p,q\right)}^{\pi_{2}}$ integral.

Inspired by the ongoing studies, we give the generalizations of the results proved in [11,15] and we prove Hermite–Hadamard-type inequalities for convex functions using the concepts of ${}^{\pi_{2}}D_{p,q}$ difference operator and ${\left(p,q\right)}^{\pi_{2}}$ integral.

The organization of this paper is as follows: In Section 2, a short explanation of the concepts of *q*-calculus and some associated works in this direction is given. In Section 3, we review the notions of (p,q)-derivatives and integrals. In Section 4, the Hermite–Hadamard-type inequalities for the (p,q)-integrals are presented. The correlation between the results presented herein and similar results in the literature are also considered. In Section 5 and Section 6, we present some new (p,q) estimates of midpoint and trapezoidal type inequalities for convex functions, respectively, and show the relationship between the results given herein and comparable results in the literature. Section 7 contains some conclusions and more directions for future research.

## 2. Preliminaries

In this portion, we review some fundamental ideas and conclusions about convex functions and *q* calculus.

A convex mapping Π:I⊂R→R is defined as:

$$\Pi \left(t\pi_{1}+\left(1-t\right)\pi_{2}\right)\leq t\Pi \left(\pi_{1}\right)+\left(1-t\right)\Pi \left(\pi_{2}\right)$$

for all $\pi_{1},\pi_{2}$ in *I* and *t* in $\left[0,1\right].$

**Definition** **1** ([38]). *A mapping* Π *defined on I has a support at $x_{0}\in I$ if there exists an affine mapping $A\left(x\right)=\Pi \left(x_{0}\right)+m\left(x-x_{0}\right)$ such that $A\left(x\right)\leq \Pi \left(x\right)$ for all x∈I. The graph of the support mapping A is called a line of support for* Π *at $x_{0}$.*

**Theorem** **1** ([38]). *A mapping $\Pi :\left(\pi_{1},\pi_{2}\right)\to R$ is convex if and only if there exists a minimum of one line of support for* Π *at each $x\in \left(\pi_{1},\pi_{2}\right)$.*

**Theorem** **2** ([39]). *If a mapping $\Pi :\left[\pi_{1},\pi_{2}\right]\to R$ is convex, then Π is also continuous on $\left(\pi_{1},\pi_{2}\right)$.*

Presently, we display a few known definitions and related inequalities in *q* calculus. We set the following notation ([4]):

$${\left[n\right]}_{q}=\frac{1-q^{n}}{1-q}=1+q+q^{2}+\ldots +q^{n-1},\;\;q\in \;\left(0,1\right).$$

The *q* Jackson integral of a mapping Π from 0 to $\pi_{2}$, given by Jackson [8], is defined as:

(2) $$\int_{0}^{\pi_{2}}\Pi \left(x\right)\begin{array}{c} d_{q}x \end{array}=\;\left(1-q\right)\pi_{2}\sum_{n=0}^{\infty }q^{n}\Pi \left(\pi_{2}q^{n}\right),\;\mathrm{where}\;0<q<1$$

provided that the sum converges absolutely. Moreover, over the interval $\left[\pi_{1},\pi_{2}\right]$, he gave the following integral of a mapping Π:

$$\int_{\pi_{1}}^{\pi_{2}}\Pi \left(x\right)\begin{array}{c} d_{q}x \end{array}=\int_{0}^{\pi_{2}}\Pi \left(x\right)\begin{array}{c} d_{q}x \end{array}-\int_{0}^{\pi_{1}}\Pi \left(x\right)\begin{array}{c} d_{q}x \end{array}.$$

**Definition** **2** ([10]). *The $q_{\pi_{1}}$-derivative of mapping $\Pi :\left[\pi_{1},\pi_{2}\right]\to R$ is defined as:*

(3) $$\begin{array}{c} {}_{\pi_{1}}D_{q}\Pi \left(x\right) \end{array}=\frac{\Pi \left(x\right)-\Pi \left(qx+\left(1-q\right)\pi_{1}\right)}{\left(1-q\right)\left(x-\pi_{1}\right)},\;x\neq \pi_{1}.$$

*For $x=\pi_{1}$, we state ${}_{\pi_{1}}D_{q}\Pi \left(\pi_{1}\right)\;=\lim_{x\to \pi_{1}}{\;}_{\pi_{1}}D_{q}\Pi \left(x\right)$ if it exists and is finite.*

**Definition** **3** ([11]). *The $q^{\pi_{2}}$ derivative of mapping $\Pi :\left[\pi_{1},\pi_{2}\right]\to R$ is given as:*

(4) $$\begin{array}{c} {}^{\pi_{2}}D_{q}\Pi \left(x\right) \end{array}=\frac{\Pi \left(qx+\left(1-q\right)\pi_{2}\right)-\Pi \left(x\right)}{\left(1-q\right)\left(\pi_{2}-x\right)},\;x\neq \pi_{2}.$$

*For $x=\pi_{2}$, we state ${}^{\pi_{2}}D_{q}\Pi \left(\pi_{2}\right)\;=\lim_{x\to \pi_{2}}{\;}^{\pi_{2}}D_{q}\Pi \left(x\right)$ if it exists and is finite.*

**Definition** **4** ([10]). *The $q_{\pi_{1}}$ definite integral of mapping $\Pi :\left[\pi_{1},\pi_{2}\right]\to R$ on $\left[\pi_{1},\pi_{2}\right]$ is defined as:*

(5) $$\int_{\pi_{1}}^{x}\Pi \left(t\right)\begin{array}{c} {}_{\pi_{1}}d_{q}t \end{array}=\;\left(1-q\right)\left(x-\pi_{1}\right)\sum_{n=0}^{\infty }q^{n}\Pi \left(q^{n}x+\left(1-q^{n}\right)\pi_{1}\right),\;x\in \left[\pi_{1},\pi_{2}\right].$$

On the other hand, the following concept of *q*-definite integral is stated by Bermudo et al. [11]:

**Definition** **5** ([11]). *The $q^{\pi_{2}}$-definite integral of mapping $\Pi :\left[\pi_{1},\pi_{2}\right]\to R$ on $\left[\pi_{1},\pi_{2}\right]$ is given as:*

(6) $$\int_{x}^{\pi_{2}}\Pi \left(t\right)\begin{array}{c} {}^{\pi_{2}}d_{q}t \end{array}=\left(1-q\right)\left(\pi_{2}-x\right)\sum_{n=0}^{\infty }q^{n}\Pi \left(q^{n}x+\left(1-q^{n}\right)\pi_{2}\right),\;x\in \left[\pi_{1},\pi_{2}\right].$$

## 3. (p,q)-Derivatives and Integrals

In this section, we review some fundamental notions and notations of $\left(p,q\right)$-calculus.

The ${\left[n\right]}_{p,q}$ is said to be (p,q) integers and is expressed as:

$${\left[n\right]}_{p,q}=\frac{p^{n}-q^{n}}{p-q}$$

with 0<q<p≤1. The ${\left[n\right]}_{p,q}!$ and $\left[\begin{array}{c} n\\ k \end{array}\right]!$ are called (p,q)-factorial and (p,q)-binomial, respectively, and expressed as:

$$\begin{array}{ccc} {\left[n\right]}_{p,q}! & = & \prod_{k=1}^{n}{\left[k\right]}_{p,q},\;n\geq 1,\;{\left[0\right]}_{p,q}!=1,\\ \left[\begin{array}{c} n\\ k \end{array}\right]! & = & \frac{{\left[n\right]}_{p,q}!}{{\left[n-k\right]}_{p,q}!{\left[k\right]}_{p,q}!}. \end{array}$$

**Definition** **6** ([12]). *The $\left(p,q\right)$-derivative of mapping $\Pi :\left[\pi_{1},\pi_{2}\right]\to R$ is given as:*

(7) $$D_{p,q}\Pi \left(x\right)=\frac{\Pi \left(px\right)-\Pi \left(qx\right)}{\left(p-q\right)x},\;x\neq 0$$

*with 0<q<p≤1.*

**Definition** **7** ([13]). *The ${\left(p,q\right)}_{\pi_{1}}$-derivative of mapping $\Pi :\left[\pi_{1},\pi_{2}\right]\to R$ is given as:*

(8) $${}_{\pi_{1}}D_{p,q}\Pi \left(x\right)=\frac{\Pi \left(px+\left(1-p\right)\pi_{1}\right)-\Pi \left(qx+\left(1-q\right)\pi_{1}\right)}{\left(p-q\right)\left(x-\pi_{1}\right)},\;x\neq \pi_{1}$$

*with 0<q<p≤1.*

For $x=\pi_{1}$, we state ${}_{\pi_{1}}D_{p,q}\Pi \left(\pi_{1}\right)=\lim_{x\to \pi_{1}}{\;}_{\pi_{1}}D_{p,q}\Pi \left(x\right)$ if it exists and is finite.

**Definition** **8** ([14]). *The ${\left(p,q\right)}^{\pi_{2}}$-derivative of mapping $\Pi :\left[\pi_{1},\pi_{2}\right]\to R$ is given as:*

(9) $${}^{\pi_{2}}D_{p,q}\Pi \left(x\right)=\frac{\Pi \left(qx+\left(1-q\right)\pi_{2}\right)-\Pi \left(px+\left(1-p\right)\pi_{2}\right)}{\left(p-q\right)\left(\pi_{2}-x\right)},\;x\neq \pi_{2}.$$

For $x=\pi_{2}$, we state ${}^{\pi_{2}}D_{p,q}\Pi \left(\pi_{2}\right)=\lim_{x\to \pi_{2}}{\;}^{\pi_{2}}D_{p,q}\Pi \left(x\right)$ if it exists and is finite.

**Remark** **1.** *It is clear that if we use p=1 in (8) and (9), then the equalities (8) and (9) reduce to (3) and (4), respectively.*

**Definition** **9** ([13]). *The definite (p,q)${}_{\pi_{1}}$-integral of mapping $\Pi :\left[\pi_{1},\pi_{2}\right]\to R$ on $\left[\pi_{1},\pi_{2}\right]$ is stated as:*

(10) $$\int_{\pi_{1}}^{x}\Pi \left(t\right){\;}_{\pi_{1}}d_{p,q}t=\left(p-q\right)\left(x-\pi_{1}\right)\sum_{n=0}^{\infty }\frac{q^{n}}{p^{n+1}}\Pi \left(\frac{q^{n}}{p^{n+1}}x+\left(1-\frac{q^{n}}{p^{n+1}}\right)\pi_{1}\right)$$

*with 0<q<p≤1.*

**Definition** **10.** *From [14], the definite (p,q)${}^{\pi_{2}}$-integral of mapping $\Pi :\left[\pi_{1},\pi_{2}\right]\to R$ on $\left[\pi_{1},\pi_{2}\right]$ is stated as:*

(11) $$\int_{x}^{\pi_{2}}\Pi \left(t\right){\;}^{\pi_{2}}d_{p,q}t=\left(p-q\right)\left(\pi_{2}-x\right)\sum_{n=0}^{\infty }\frac{q^{n}}{p^{n+1}}\Pi \left(\frac{q^{n}}{p^{n+1}}x+\left(1-\frac{q^{n}}{p^{n+1}}\right)\pi_{2}\right)$$

*with 0<q<p≤1.*

**Remark** **2.** *It is evident that if we pick p=1 in (10) and (11), then the equalities (10) and (11) change into (5) and (6), respectively.*

**Remark** **3.** *If we take $\pi_{1}=0$ and $x=\pi_{2}=1$ in (10), then we have*

$$\int_{0}^{1}\Pi \left(t\right){\;}_{0}d_{p,q}t=\left(p-q\right)\sum_{n=0}^{\infty }\frac{q^{n}}{p^{n+1}}\Pi \left(\frac{q^{n}}{p^{n+1}}\right).$$

*Similarly, by taking $x=\pi_{1}=0$ and $\pi_{2}=1$ in (11), then we obtain that*

$$\int_{0}^{1}\Pi \left(t\right){\;}^{1}d_{p,q}t=\left(p-q\right)\sum_{n=0}^{\infty }\frac{q^{n}}{p^{n+1}}\Pi \left(1-\frac{q^{n}}{p^{n+1}}\right).$$

In [36], Kunt et al. proved the following HH-type inequalities for convex functions via (p,q)${}_{\pi_{1}}$ integral:

**Theorem** **3.** *For a convex mapping $\Pi :\left[\pi_{1},\pi_{2}\right]\to R$ which is differentiable on $\left[\pi_{1},\pi_{2}\right],$ the following inequalities hold for ${\left(p,q\right)}_{\pi_{1}}$ integral:*

(12) $$\Pi \left(\frac{q\pi_{1}+p\pi_{2}}{{\left[2\right]}_{p,q}}\right)\leq \frac{1}{p\left(\pi_{2}-\pi_{1}\right)}\int_{\pi_{1}}^{p\pi_{2}+\left(1-p\right)\pi_{1}}\Pi \left(x\right){\;}_{\pi_{1}}d_{p,q}x\leq \frac{q\Pi \left(\pi_{1}\right)+p\Pi \left(\pi_{2}\right)}{{\left[2\right]}_{p,q}}$$

*where 0<q<p≤1.*

**Lemma** **1.** *We have the following equalities*

$$\int_{\pi_{1}}^{\pi_{2}}{\left(\pi_{2}-x\right)}^{\alpha }{\;}^{\pi_{2}}d_{p,q}x=\frac{{\left(\pi_{2}-\pi_{1}\right)}^{\alpha +1}}{{\left[\alpha +1\right]}_{p,q}}$$

$$\int_{\pi_{1}}^{\pi_{2}}{\left(x-\pi_{1}\right)}^{\alpha }{\;}_{\pi_{1}}d_{p,q}x=\frac{{\left(\pi_{2}-\pi_{1}\right)}^{\alpha +1}}{{\left[\alpha +1\right]}_{p,q}}$$

*where α∈R−{−1}.*

**Proof.**  From Definition 10, we have

$$\begin{array}{ccc} \int_{\pi_{1}}^{\pi_{2}}{\left(\pi_{2}-x\right)}^{\alpha }{\;}^{\pi_{2}}d_{p,q}x & = & \left(p-q\right)\left(\pi_{2}-\pi_{1}\right)\sum_{n=0}^{\infty }\frac{q^{n}}{p^{n+1}}{\left(\pi_{2}-\left(\frac{q^{n}}{p^{n+1}}\pi_{1}+\left(1-\frac{q^{n}}{p^{n+1}}\right)\pi_{2}\right)\right)}^{\alpha }\\ & = & \left(p-q\right)\left(\pi_{2}-\pi_{1}\right)\sum_{n=0}^{\infty }\frac{q^{n}}{p^{n+1}}{\left(\frac{q^{n}}{p^{n+1}}\left(\pi_{2}-\pi_{1}\right)\right)}^{\alpha }\\ & = & \left(p-q\right){\left(\pi_{2}-\pi_{1}\right)}^{\alpha +1}\sum_{n=0}^{\infty }\frac{1}{p^{\alpha +1}}{\left(\frac{q}{p}\right)}^{n\left(\alpha +1\right)}\\ & = & \frac{{\left(\pi_{2}-\pi_{1}\right)}^{\alpha +1}}{{\left[\alpha +1\right]}_{p,q}}. \end{array}$$

Similarly, we can compute the second integral by using the Definition 9. □

## 4. New HH Type Inequalities for Post-Quantum Integrals

In this section, we give a new variant of (p,q)-HH inequality for convex functions. It is also shown that the results presented here are a generalization of some existing results in the literature.

**Theorem** **4.** *For a convex mapping $\Pi :\left[\pi_{1},\pi_{2}\right]\to R$, which is differentiable on $\left[\pi_{1},\pi_{2}\right],$ the following inequalities hold for ${\left(p,q\right)}^{\pi_{2}}$ integral:*

(13) $$\Pi \left(\frac{p\pi_{1}+q\pi_{2}}{{\left[2\right]}_{p,q}}\right)\leq \frac{1}{p\left(\pi_{2}-\pi_{1}\right)}\int_{p\pi_{1}+\left(1-p\right)\pi_{2}}^{\pi_{2}}\Pi \left(x\right){\;}^{\pi_{2}}d_{p,q}x\leq \frac{p\Pi \left(\pi_{1}\right)+q\Pi \left(\pi_{2}\right)}{{\left[2\right]}_{p,q}}$$

*where 0<q<p≤1.*

**Figure 1:**
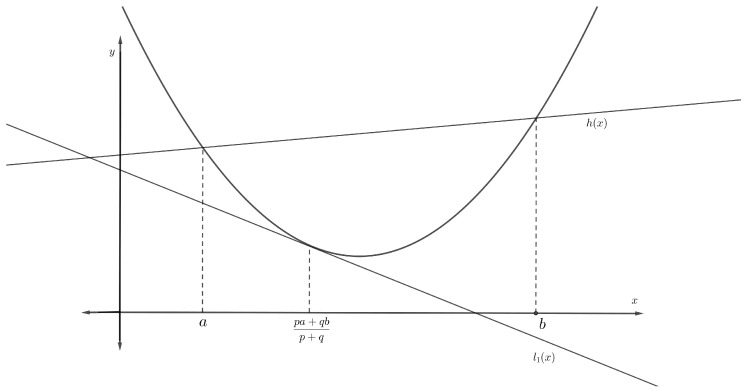
Tangent line at the point $\frac{pa+qb}{p+q}=\frac{p\pi_{1}+q\pi_{2}}{p+q}$ of the convex function Π and chord line.

**Proof.**  According to the given hypothesis, Π is differentiable on $\left[\pi_{1},\pi_{2}\right]$, so there exists a tangent line for the function Π at the point $\frac{p\pi_{1}+q\pi_{2}}{{\left[2\right]}_{p,q}}.$ This tangent line can be indicated as a function $l_{1}\left(x\right)=\Pi \left(\frac{p\pi_{1}+q\pi_{2}}{{\left[2\right]}_{p,q}}\right)+\Pi^{\prime }\left(\frac{p\pi_{1}+q\pi_{2}}{{\left[2\right]}_{p,q}}\right)\left(x-\frac{p\pi_{1}+q\pi_{2}}{{\left[2\right]}_{p,q}}\right).$ Since Π is convex on $\left[\pi_{1},\pi_{2}\right]$, the following inequality (see Figure 1) holds for all *x* in $\left[\pi_{1},\pi_{2}\right]:$

(14) $$l_{1}\left(x\right)=\Pi \left(\frac{p\pi_{1}+q\pi_{2}}{{\left[2\right]}_{p,q}}\right)+\Pi^{\prime }\left(\frac{p\pi_{1}+q\pi_{2}}{{\left[2\right]}_{p,q}}\right)\left(x-\frac{p\pi_{1}+q\pi_{2}}{{\left[2\right]}_{p,q}}\right)\leq \Pi \left(x\right)$$

${\left(p,q\right)}^{\pi_{2}}$-integrating inequality (14) with respect to *x* over $\left[p\pi_{1}+\left(1-p\right)\pi_{2},\pi_{2}\right]$, we find that

$$\begin{array}{ccc} & & \int_{p\pi_{1}+\left(1-p\right)\pi_{2}}^{\pi_{2}}l_{1}\left(x\right){\;}^{\pi_{2}}d_{p,q}x\\ & = & \int_{p\pi_{1}+\left(1-p\right)\pi_{2}}^{\pi_{2}}\left[\Pi \left(\frac{p\pi_{1}+q\pi_{2}}{{\left[2\right]}_{p,q}}\right)+\Pi^{\prime }\left(\frac{p\pi_{1}+q\pi_{2}}{{\left[2\right]}_{p,q}}\right)\left(x-\frac{p\pi_{1}+q\pi_{2}}{{\left[2\right]}_{p,q}}\right)\right]{\;}^{\pi_{2}}d_{p,q}x\\ & = & p\left(\pi_{2}-\pi_{1}\right)\Pi \left(\frac{p\pi_{1}+q\pi_{2}}{{\left[2\right]}_{p,q}}\right)+\Pi^{\prime }\left(\frac{p\pi_{1}+q\pi_{2}}{{\left[2\right]}_{p,q}}\right)\int_{p\pi_{1}+\left(1-p\right)\pi_{2}}^{\pi_{2}}x{\;}^{\pi_{2}}d_{p,q}x\\ & & -p\left(\pi_{2}-\pi_{1}\right)\frac{p\pi_{1}+q\pi_{2}}{{\left[2\right]}_{p,q}}\Pi^{\prime }\left(\frac{p\pi_{1}+q\pi_{2}}{{\left[2\right]}_{p,q}}\right)\\ & = & p\left(\pi_{2}-\pi_{1}\right)\Pi \left(\frac{p\pi_{1}+q\pi_{2}}{{\left[2\right]}_{p,q}}\right)-p\left(\pi_{2}-\pi_{1}\right)\frac{p\pi_{1}+q\pi_{2}}{{\left[2\right]}_{p,q}}\Pi^{\prime }\left(\frac{p\pi_{1}+q\pi_{2}}{{\left[2\right]}_{p,q}}\right)\\ & & +\Pi^{\prime }\left(\frac{p\pi_{1}+q\pi_{2}}{{\left[2\right]}_{p,q}}\right)\left[\left(p-q\right)p\left(\pi_{2}-\pi_{1}\right)\sum_{n=0}^{\infty }\frac{q^{n}}{p^{n+1}}\left(\frac{q^{n}}{p^{n+1}}\left(p\pi_{1}+\left(1-p\right)\pi_{2}\right)+\left(1-\frac{q^{n}}{p^{n+1}}\right)\pi_{2}\right)\right]\\ & = & p\left(\pi_{2}-\pi_{1}\right)\Pi \left(\frac{p\pi_{1}+q\pi_{2}}{{\left[2\right]}_{p,q}}\right)-p\left(\pi_{2}-\pi_{1}\right)\frac{p\pi_{1}+q\pi_{2}}{{\left[2\right]}_{p,q}}\Pi^{\prime }\left(\frac{p\pi_{1}+q\pi_{2}}{{\left[2\right]}_{p,q}}\right)\\ & & +\Pi^{\prime }\left(\frac{p\pi_{1}+q\pi_{2}}{{\left[2\right]}_{p,q}}\right)\left[\left(p-q\right)\left(\pi_{2}-\pi_{1}\right)\sum_{n=0}^{\infty }\left(\frac{q^{n}}{p^{n+1}}\pi_{2}-\frac{q^{2n}}{p^{2n+1}}\left(\pi_{2}-\pi_{1}\right)\right)\right]\\ & = & p\left(\pi_{2}-\pi_{1}\right)\Pi \left(\frac{p\pi_{1}+q\pi_{2}}{{\left[2\right]}_{p,q}}\right)-p\left(\pi_{2}-\pi_{1}\right)\frac{p\pi_{1}+q\pi_{2}}{{\left[2\right]}_{p,q}}\Pi^{\prime }\left(\frac{p\pi_{1}+q\pi_{2}}{{\left[2\right]}_{p,q}}\right)\\ & & +p\left(\pi_{2}-\pi_{1}\right)\frac{p\pi_{1}+q\pi_{2}}{{\left[2\right]}_{p,q}}\Pi^{\prime }\left(\frac{p\pi_{1}+q\pi_{2}}{{\left[2\right]}_{p,q}}\right)\\ & = & p\left(\pi_{2}-\pi_{1}\right)\Pi \left(\frac{p\pi_{1}+q\pi_{2}}{{\left[2\right]}_{p,q}}\right)\leq \int_{p\pi_{1}+\left(1-p\right)\pi_{2}}^{\pi_{2}}\Pi \left(x\right){\;}^{\pi_{2}}d_{p,q}x \end{array}$$

where the first inequality in (13) is derived. We also have to show the second inequality in (13). According to the given hypothesis, Π is convex on $\left[\pi_{1},\pi_{2}\right]$, so $\Pi \left(x\right)\leq h\left(x\right)$, where $h\left(x\right)$ is a secant line that connects the points ($\pi_{1},\Pi \left(\pi_{1}\right)$) and $\left(\pi_{2},\Pi \left(\pi_{2}\right)\right)$, expressed as:

(15) $$\Pi \left(x\right)\leq h\left(x\right)=\Pi \left(\pi_{2}\right)+\frac{\Pi \left(\pi_{2}\right)-\Pi \left(\pi_{1}\right)}{\pi_{2}-\pi_{1}}\left(x-\pi_{2}\right)$$

for all *x* in $\left[\pi_{1},\pi_{2}\right]$ (see Figure 1). ${\left(p,q\right)}^{\pi_{2}}$-integrating inequality (15) with respect to *x* over $\left[p\pi_{1}+\left(1-p\right)\pi_{2},\pi_{2}\right]$, we obtain the following

(16) $$\begin{array}{ccc} & & \int_{p\pi_{1}+\left(1-p\right)\pi_{2}}^{\pi_{2}}\Pi \left(x\right){\;}^{\pi_{2}}d_{p,q}x\\ & \leq & p\left(\pi_{2}-\pi_{1}\right)\Pi \left(\pi_{2}\right)-p\pi_{2}\left(\pi_{2}-\pi_{1}\right)\frac{\Pi \left(\pi_{2}\right)-\Pi \left(\pi_{1}\right)}{\pi_{2}-\pi_{1}}+\frac{\Pi \left(\pi_{2}\right)-\Pi \left(\pi_{1}\right)}{\pi_{2}-\pi_{1}}\int_{p\pi_{1}+\left(1-p\right)\pi_{2}}^{\pi_{2}}x{\;}^{\pi_{2}}d_{p,q}x\\ & = & p\left(\pi_{2}-\pi_{1}\right)\Pi \left(\pi_{2}\right)-\frac{\Pi \left(\pi_{2}\right)-\Pi \left(\pi_{1}\right)}{\pi_{2}-\pi_{1}}\left(\frac{p^{2}{\left(\pi_{2}-\pi_{1}\right)}^{2}}{{\left[2\right]}_{p,q}}\right)\\ & = & p\left(\pi_{2}-\pi_{1}\right)\Pi \left(\pi_{2}\right)-\left(\Pi \left(\pi_{2}\right)-\Pi \left(\pi_{1}\right)\right)\frac{p^{2}\left(\pi_{2}-\pi_{1}\right)}{{\left[2\right]}_{p,q}}\\ & = & p\left(\pi_{2}-\pi_{1}\right)\frac{p\Pi \left(\pi_{1}\right)+q\Pi \left(\pi_{2}\right)}{{\left[2\right]}_{p,q}} \end{array}$$

where the last inequality in (13) is obtained. Thus, the proof is completed. □

**Example** **1.** *For a convex mapping $\Pi \left(x\right)=x^{2}$ and $\pi_{1}=0$, $\pi_{2}=1$, $p=\frac{3}{4}$, and $q=\frac{1}{2}$. From inequality (13), we have*

$$\Pi \left(\frac{p\pi_{1}+q\pi_{2}}{{\left[2\right]}_{p,q}}\right)=0.16,$$

$$\frac{1}{p\left(\pi_{2}-\pi_{1}\right)}\int_{p\pi_{1}+\left(1-p\right)\pi_{2}}^{\pi_{2}}\Pi \left(x\right){\;}^{\pi_{2}}d_{p,q}x=\frac{4}{3}\int_{\frac{1}{4}}^{1}x^{2}{\;}^{1}d_{\frac{3}{4},\frac{1}{2}}x=0.2736,$$

*and*

$$\frac{p\Pi \left(\pi_{1}\right)+q\Pi \left(\pi_{2}\right)}{{\left[2\right]}_{p,q}}=0.4.$$

*Thus,*

0.16<0.2736<0.4

*which shows that the inequality (13) is valid.*

**Corollary** **1.** *For a convex mapping $\Pi :\left[\pi_{1},\pi_{2}\right]\to R,$ the following inequality holds: *

(17) $$\begin{array}{ccc} \Pi \left(\frac{\pi_{1}+\pi_{2}}{2}\right) & \leq & \frac{1}{2p\left(\pi_{2}-\pi_{1}\right)}\left[\int_{\pi_{1}}^{p\pi_{2}+\left(1-p\right)\pi_{1}}\Pi \left(x\right){\;}_{\pi_{1}}d_{p,q}x+\int_{p\pi_{1}+\left(1-p\right)\pi_{2}}^{\pi_{2}}\Pi \left(x\right){\;}^{\pi_{2}}d_{p,q}x\right]\\ & \leq & \frac{\Pi \left(\pi_{1}\right)+\Pi \left(\pi_{2}\right)}{2} \end{array}$$

*where 0<q<p≤1.*

**Proof.**  From inequalities (12) and (13), one can easily obtain the resultant inequality (17). □

In the subsequent theorem, we give an alternative proof of the double inequality (13) without using the condition of differentiability on Π.

**Theorem** **5.** *For a convex mapping Π:I→R on I and $\pi_{1},\pi_{2}\in I^{\circ }\;$with $\pi_{1}<\pi_{2}$, the double inequality (13) holds for 0<q<p≤1.*

**Proof.**  According to the given hypothesis that Π is convex on *I*, by Theorem 2, Π is continuous on $\left[\pi_{1},\pi_{2}\right]$. By means of Theorem 1, there exists a minimum of one line of support for Π at each $x_{0}\in \left(\pi_{1},\pi_{2}\right)$. Since $x_{0}=\frac{p\pi_{1}+q\pi_{2}}{{\left[2\right]}_{p,q}}$, from the definition 1

(18) $$k\left(x\right)=\Pi \left(\frac{p\pi_{1}+q\pi_{2}}{{\left[2\right]}_{p,q}}\right)+m\left(x-\frac{p\pi_{1}+q\pi_{2}}{{\left[2\right]}_{p,q}}\right)\leq \Pi \left(x\right)$$

for all $x\in \left[\pi_{1},\pi_{2}\right]$ and some $m\in \left[\Pi_{-}^{\prime }\left(\frac{p\pi_{1}+q\pi_{2}}{{\left[2\right]}_{p,q}}\right),\Pi_{+}^{\prime }\left(\frac{p\pi_{1}+q\pi_{2}}{{\left[2\right]}_{p,q}}\right)\right]$. If the strategy that was used in the proof of Theorem 4 is applied and taking into account the inequality (18), the desired inequality (13) can be found. Thus, the proof is accomplished. □

**Remark** **4.** *If we consider p=1 in Theorems 4 and 5, then Theorem 4 and 5 reduces to [11] (Theorem 12).*

**Remark** **5.** *If we adopt p=1 and $q\to 1^{-}$ in Theorems 4 and 5, then we retake the well-known HH inequality for convex functions.*

**Theorem** **6.** *For a convex mapping $\Pi :\left[\pi_{1},\pi_{2}\right]\to R$, which is differentiable on $\left[\pi_{1},\pi_{2}\right]$, the following inequalities hold for ${\left(p,q\right)}^{\pi_{2}}$-integral:*

(19) $$\begin{array}{ccc} & & \Pi \left(\frac{q\pi_{1}+p\pi_{2}}{{\left[2\right]}_{p,q}}\right)+\frac{\left(p-q\right)\left(\pi_{2}-\pi_{1}\right)}{{\left[2\right]}_{p,q}}\Pi^{\prime }\left(\frac{q\pi_{1}+p\pi_{2}}{{\left[2\right]}_{p,q}}\right)\\ & \leq & \frac{1}{p\left(\pi_{2}-\pi_{1}\right)}\int_{p\pi_{1}+\left(1-p\right)\pi_{2}}^{\pi_{2}}\Pi \left(x\right){\;}^{\pi_{2}}d_{p,q}x\\ & \leq & \frac{p\Pi \left(\pi_{1}\right)+q\Pi \left(\pi_{2}\right)}{{\left[2\right]}_{p,q}} \end{array}$$

*where 0<q<p≤1.*

**Figure 2:**
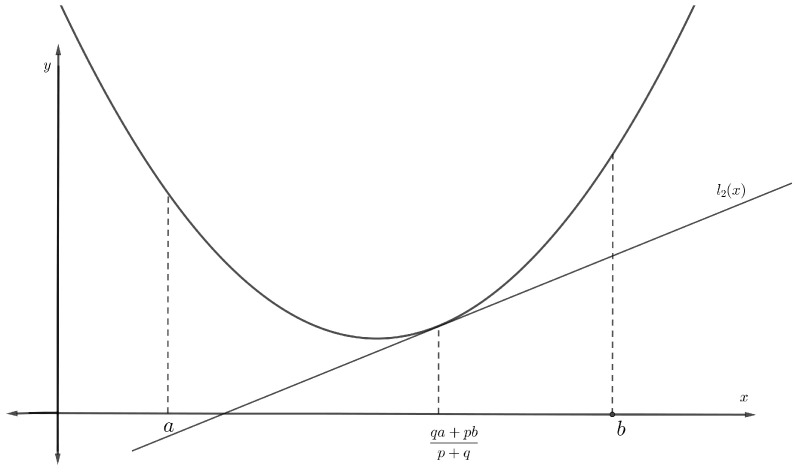
Tangent line at the point $\frac{qa+pb}{p+q}=\frac{q\pi_{1}+p\pi_{2}}{p+q}$ of the convex function Π.

**Proof.**  According to the given hypothesis, Π is differentiable on $\left[\pi_{1},\pi_{2}\right],$ so there exists a tangent line for the function Π at the point $\frac{q\pi_{1}+p\pi_{2}}{{\left[2\right]}_{p,q}}.$ This tangent line can be indicated as a function $l_{2}\left(x\right)=\Pi \left(\frac{q\pi_{1}+p\pi_{2}}{{\left[2\right]}_{p,q}}\right)+\Pi^{\prime }\left(\frac{q\pi_{1}+p\pi_{2}}{{\left[2\right]}_{p,q}}\right)\left(x-\frac{q\pi_{1}+p\pi_{2}}{{\left[2\right]}_{p,q}}\right).$ Since Π is convex on $\left[\pi_{1},\pi_{2}\right]$, then the following inequality (see Figure 2) holds for all *x* in $\left[\pi_{1},\pi_{2}\right]:$

(20) $$l_{2}\left(x\right)=\Pi \left(\frac{q\pi_{1}+p\pi_{2}}{{\left[2\right]}_{p,q}}\right)+\Pi^{\prime }\left(\frac{q\pi_{1}+p\pi_{2}}{{\left[2\right]}_{p,q}}\right)\left(x-\frac{q\pi_{1}+p\pi_{2}}{{\left[2\right]}_{p,q}}\right)\leq \Pi \left(x\right).$$

${\left(p,q\right)}^{\pi_{2}}$-integrating inequality (20) with respect to *x* over $\left[p\pi_{1}+\left(1-p\right)\pi_{2},\pi_{2}\right]$, we obtain that

(21) $$\begin{array}{ccc} & & \int_{p\pi_{1}+\left(1-p\right)\pi_{2}}^{\pi_{2}}l_{2}\left(x\right){\;}^{\pi_{2}}d_{p,q}x\\ & = & \int_{p\pi_{1}+\left(1-p\right)\pi_{2}}^{\pi_{2}}\left[\Pi \left(\frac{q\pi_{1}+p\pi_{2}}{{\left[2\right]}_{p,q}}\right)+\Pi^{\prime }\left(\frac{q\pi_{1}+p\pi_{2}}{{\left[2\right]}_{p,q}}\right)\left(x-\frac{q\pi_{1}+p\pi_{2}}{{\left[2\right]}_{p,q}}\right)\right]{\;}^{\pi_{2}}d_{p,q}x\\ & = & p\left(\pi_{2}-\pi_{1}\right)\Pi \left(\frac{q\pi_{1}+p\pi_{2}}{{\left[2\right]}_{p,q}}\right)+\Pi^{\prime }\left(\frac{q\pi_{1}+p\pi_{2}}{{\left[2\right]}_{p,q}}\right)\int_{p\pi_{1}+\left(1-p\right)\pi_{2}}^{\pi_{2}}x{\;}^{\pi_{2}}d_{p,q}x\\ & & -p\left(\pi_{2}-\pi_{1}\right)\frac{q\pi_{1}+p\pi_{2}}{{\left[2\right]}_{p,q}}\Pi^{\prime }\left(\frac{q\pi_{1}+p\pi_{2}}{{\left[2\right]}_{p,q}}\right)\\ & = & p\left(\pi_{2}-\pi_{1}\right)\Pi \left(\frac{q\pi_{1}+p\pi_{2}}{{\left[2\right]}_{p,q}}\right)-p\left(\pi_{2}-\pi_{1}\right)\frac{q\pi_{1}+p\pi_{2}}{{\left[2\right]}_{p,q}}\Pi^{\prime }\left(\frac{q\pi_{1}+p\pi_{2}}{{\left[2\right]}_{p,q}}\right)\\ & & +\Pi^{\prime }\left(\frac{q\pi_{1}+p\pi_{2}}{{\left[2\right]}_{p,q}}\right)\\ & & \times \left[\left(p-q\right)p\left(\pi_{2}-\pi_{1}\right)\sum_{n=0}^{\infty }\frac{q^{n}}{p^{n+1}}\left(\frac{q^{n}}{p^{n+1}}\left(p\pi_{1}+\left(1-p\right)\pi_{2}\right)+\left(1-\frac{q^{n}}{p^{n+1}}\right)\pi_{2}\right)\right]\\ & = & p\left(\pi_{2}-\pi_{1}\right)\Pi \left(\frac{q\pi_{1}+p\pi_{2}}{{\left[2\right]}_{p,q}}\right)-p\left(\pi_{2}-\pi_{1}\right)\frac{q\pi_{1}+p\pi_{2}}{{\left[2\right]}_{p,q}}\Pi^{\prime }\left(\frac{q\pi_{1}+p\pi_{2}}{{\left[2\right]}_{p,q}}\right)\\ & & +\Pi^{\prime }\left(\frac{q\pi_{1}+p\pi_{2}}{{\left[2\right]}_{p,q}}\right)\left[\left(p-q\right)\left(\pi_{2}-\pi_{1}\right)\sum_{n=0}^{\infty }\left(\frac{q^{n}}{p^{n+1}}\pi_{2}-\frac{q^{2n}}{p^{2n+1}}\left(\pi_{2}-\pi_{1}\right)\right)\right]\\ & = & p\left(\pi_{2}-\pi_{1}\right)\Pi \left(\frac{p\pi_{1}+q\pi_{2}}{{\left[2\right]}_{p,q}}\right)-p\left(\pi_{2}-\pi_{1}\right)\frac{q\pi_{1}+p\pi_{2}}{{\left[2\right]}_{p,q}}\Pi^{\prime }\left(\frac{q\pi_{1}+p\pi_{2}}{{\left[2\right]}_{p,q}}\right)\\ & & +p\left(\pi_{2}-\pi_{1}\right)\frac{p\pi_{1}+q\pi_{2}}{{\left[2\right]}_{p,q}}\Pi^{\prime }\left(\frac{q\pi_{1}+p\pi_{2}}{{\left[2\right]}_{p,q}}\right)\\ & = & p\left(\pi_{2}-\pi_{1}\right)\Pi \left(\frac{p\pi_{1}+q\pi_{2}}{{\left[2\right]}_{p,q}}\right)+\frac{p\left(p-q\right){\left(\pi_{2}-\pi_{1}\right)}^{2}}{{\left[2\right]}_{p,q}}\Pi^{\prime }\left(\frac{q\pi_{1}+p\pi_{2}}{{\left[2\right]}_{p,q}}\right)\\ & \leq & \int_{p\pi_{1}+\left(1-p\right)\pi_{2}}^{\pi_{2}}\Pi \left(x\right){\;}^{\pi_{2}}d_{p,q}x. \end{array}$$

From (16) and (21), we obtain the desired result (19). Thus, the proof is finished. □

**Theorem** **7.** *Let $\Pi :\left[\pi_{1},\pi_{2}\right]\to R$ be a convex differentiable function on $\left[\pi_{1},\pi_{2}\right].$ Then, the following inequalities hold for ${\left(p,q\right)}^{\pi_{2}}$ integral:*

(22) $$\max\left\{A_{1},A_{2}\right\}\leq \frac{1}{p\left(\pi_{2}-\pi_{1}\right)}\int_{p\pi_{1}+\left(1-p\right)\pi_{2}}^{\pi_{2}}\Pi \left(x\right){\;}^{\pi_{2}}d_{p,q}x\leq \frac{p\Pi \left(\pi_{1}\right)+q\Pi \left(\pi_{2}\right)}{{\left[2\right]}_{p,q}}$$

*where*

$$\begin{array}{ccc} A_{1} & = & \Pi \left(\frac{p\pi_{1}+q\pi_{2}}{{\left[2\right]}_{p,q}}\right),\\ A_{2} & = & \Pi \left(\frac{q\pi_{1}+p\pi_{2}}{{\left[2\right]}_{p,q}}\right)+\frac{\left(p-q\right)\left(\pi_{2}-\pi_{1}\right)}{{\left[2\right]}_{p,q}}\Pi^{\prime }\left(\frac{q\pi_{1}+p\pi_{2}}{{\left[2\right]}_{p,q}}\right) \end{array}$$

*and 0<q<p≤1.*

**Proof.**  From (13) and (19), we have required double inequality (22). Thus, the proof is ended. □

## 5. Midpoint-Type Inequalities through (p,q)${}^{\pi_{2}}$ Integral

In this section, we give some new midpoint-type inequalities by using the $\left(p,q\right)$-derivative and integral.

To prove the main results of this section, we need the following crucial lemma.

**Lemma** **2.** *Let $\Pi :\left[\pi_{1},\pi_{2}\right]\to R$ be a differentiable function on $\left(\pi_{1},\pi_{2}\right)$. If ${}^{\pi_{2}}D_{p,q}\Pi$ is continuous and integrable on $\left[\pi_{1},\pi_{2}\right]$, then we have the following identity:*

(23) $$\begin{array}{ccc} & & \left(\pi_{2}-\pi_{1}\right)\left[\int_{0}^{\frac{p}{{\left[2\right]}_{p,q}}}qt{\;}^{\pi_{2}}D_{p,q}\Pi \left(t\pi_{1}+\left(1-t\right)\pi_{2}\right)d_{p,q}t\right.\\ & & \left.+\int_{\frac{p}{{\left[2\right]}_{p,q}}}^{1}\left(qt-1\right){\;}^{\pi_{2}}D_{p,q}\Pi \left(t\pi_{1}+\left(1-t\right)\pi_{2}\right)d_{p,q}t\right]\\ & = & \int_{\pi_{1}p+\left(1-p\right)\pi_{2}}^{\pi_{2}}\Pi \left(x\right){\;}^{\pi_{2}}d_{p,q}x-\Pi \left(\frac{p\pi_{1}+q\pi_{2}}{{\left[2\right]}_{p,q}}\right) \end{array}$$

*where 0<q<p≤1.*

**Proof.**  From Definition 8, we have

(24) $${}^{\pi_{2}}D_{p,q}\Pi \left(t\pi_{1}+\left(1-t\right)\pi_{2}\right)=\frac{\Pi \left(qt\pi_{1}+\left(1-qt\right)\pi_{2}\right)-\Pi \left(pt\pi_{1}+\left(1-pt\right)\pi_{2}\right)}{t\left(\pi_{2}-\pi_{1}\right)\left(p-q\right)}.$$

From the left side of equality (23), we have

(25) $$\begin{array}{ccc} & & \left(\pi_{2}-\pi_{1}\right)\left[\int_{0}^{\frac{p}{{\left[2\right]}_{p,q}}}qt{\;}^{\pi_{2}}D_{p,q}\Pi \left(t\pi_{1}+\left(1-t\right)\pi_{2}\right)d_{p,q}t\right.\\ & & \left.+\int_{\frac{p}{{\left[2\right]}_{p,q}}}^{1}\left(qt-1\right){\;}^{\pi_{2}}D_{p,q}\Pi \left(t\pi_{1}+\left(1-t\right)\pi_{2}\right)d_{p,q}t\right]\\ & = & \left(\pi_{2}-\pi_{1}\right)\left[\int_{0}^{\frac{p}{{\left[2\right]}_{p,q}}}{\;}^{\pi_{2}}D_{p,q}\Pi \left(t\pi_{1}+\left(1-t\right)\pi_{2}\right)d_{p,q}t+\int_{0}^{1}qt{\;}^{\pi_{2}}D_{p,q}\Pi \left(t\pi_{1}+\left(1-t\right)\pi_{2}\right)d_{p,q}t\right.\\ & & \left.-\int_{0}^{1}{\;}^{\pi_{2}}D_{p,q}\Pi \left(t\pi_{1}+\left(1-t\right)\pi_{2}\right)d_{p,q}t\right]. \end{array}$$

By the equality (11), we have

(26) $$\begin{array}{ccc} & & \int_{0}^{\frac{p}{{\left[2\right]}_{p,q}}}{\;}^{\pi_{2}}D_{p,q}\Pi \left(t\pi_{1}+\left(1-t\right)\pi_{2}\right)d_{p,q}t\\ & = & \frac{1}{\left(\pi_{2}-\pi_{1}\right)\left(p-q\right)}\int_{0}^{\frac{p}{{\left[2\right]}_{p,q}}}\frac{\Pi \left(qt\pi_{1}+\left(1-qt\right)\pi_{2}\right)-\Pi \left(pt\pi_{1}+\left(1-pt\right)\pi_{2}\right)}{t}d_{p,q}t\\ & = & \frac{1}{\pi_{2}-\pi_{1}}\left[\sum_{n=0}^{\infty }\Pi \left(\frac{p}{{\left[2\right]}_{p,q}}\frac{q^{n+1}}{p^{n+1}}\pi_{1}+\left(1-\frac{p}{{\left[2\right]}_{p,q}}\frac{q^{n+1}}{p^{n+1}}\right)\pi_{2}\right)\right.\\ & & \left.-\sum_{n=0}^{\infty }\Pi \left(\frac{p}{{\left[2\right]}_{p,q}}\frac{q^{n}}{p^{n}}\pi_{1}+\left(1-\frac{p}{{\left[2\right]}_{p,q}}\frac{q^{n}}{p^{n}}\right)\pi_{2}\right)\right]\\ & = & \frac{\Pi \left(\pi_{2}\right)}{\pi_{2}-\pi_{1}}-\frac{1}{\pi_{2}-\pi_{1}}\Pi \left(\frac{p\pi_{1}+q\pi_{2}}{{\left[2\right]}_{p,q}}\right), \end{array}$$

(27) $$\begin{array}{ccc} & & \int_{0}^{1}{\;}^{\pi_{2}}D_{p,q}\Pi \left(t\pi_{1}+\left(1-t\right)\pi_{2}\right)d_{p,q}t\\ & = & \frac{1}{\left(\pi_{2}-\pi_{1}\right)\left(p-q\right)}\int_{0}^{1}\frac{\Pi \left(qt\pi_{1}+\left(1-qt\right)\pi_{2}\right)-\Pi \left(pt\pi_{1}+\left(1-pt\right)\pi_{2}\right)}{t}d_{p,q}t\\ & = & \frac{1}{\pi_{2}-\pi_{1}}\left[\sum_{n=0}^{\infty }\Pi \left(\frac{q^{n+1}}{p^{n+1}}\pi_{1}+\left(1-\frac{q^{n+1}}{p^{n+1}}\right)\pi_{2}\right)-\sum_{n=0}^{\infty }\Pi \left(\frac{q^{n}}{p^{n}}\pi_{1}+\left(1-\frac{q^{n}}{p^{n}}\right)\pi_{2}\right)\right]\\ & = & \frac{\Pi \left(\pi_{2}\right)-\Pi \left(\pi_{1}\right)}{\pi_{2}-\pi_{1}} \end{array}$$

and

(28) $$\begin{array}{ccc} & & \int_{0}^{1}t{\;}^{\pi_{2}}D_{p,q}\Pi \left(t\pi_{1}+\left(1-t\right)\pi_{2}\right)d_{p,q}t\\ & = & \frac{1}{\left(\pi_{2}-\pi_{1}\right)\left(p-q\right)}\int_{0}^{1}\left[\Pi \left(qt\pi_{1}+\left(1-qt\right)\pi_{2}\right)-\Pi \left(pt\pi_{1}+\left(1-pt\right)\pi_{2}\right)\right]d_{p,q}t\\ & = & \frac{1}{\pi_{2}-\pi_{1}}\left[\sum_{n=0}^{\infty }\frac{q^{n}}{p^{n+1}}\Pi \left(\frac{q^{n+1}}{p^{n+1}}\pi_{1}+\left(1-\frac{q^{n+1}}{p^{n+1}}\right)\pi_{2}\right)-\sum_{n=0}^{\infty }\frac{q^{n}}{p^{n+1}}\Pi \left(\frac{q^{n}}{p^{n}}\pi_{1}+\left(1-\frac{q^{n}}{p^{n}}\right)\pi_{2}\right)\right]\\ & = & \frac{1}{\pi_{2}-\pi_{1}}\left[\frac{1}{q}\sum_{n=0}^{\infty }\frac{q^{n+1}}{p^{n+1}}\Pi \left(\frac{q^{n+1}}{p^{n+1}}\pi_{1}+\left(1-\frac{q^{n+1}}{p^{n+1}}\right)\pi_{2}\right)-\frac{1}{p}\sum_{n=0}^{\infty }\frac{q^{n}}{p^{n}}\Pi \left(\frac{q^{n}}{p^{n}}\pi_{1}+\left(1-\frac{q^{n}}{p^{n}}\right)\pi_{2}\right)\right]\\ & = & \frac{1}{\pi_{2}-\pi_{1}}\left[\left(\frac{1}{q}-\frac{1}{p}\right)\sum_{n=0}^{\infty }\frac{q^{n}}{p^{n}}\Pi \left(\frac{q^{n}}{p^{n}}\pi_{1}+\left(1-\frac{q^{n}}{p^{n}}\right)\pi_{2}\right)-\frac{1}{q}\Pi \left(\pi_{1}\right)\right]\\ & = & \frac{1}{\pi_{2}-\pi_{1}}\left[\frac{p-q}{pq}\sum_{n=0}^{\infty }\frac{q^{n}}{p^{n}}\Pi \left(\frac{q^{n}}{p^{n}}\pi_{1}+\left(1-\frac{q^{n}}{p^{n}}\right)\pi_{2}\right)-\frac{1}{q}\Pi \left(\pi_{1}\right)\right]\\ & = & \frac{1}{\pi_{2}-\pi_{1}}\left[\frac{1}{pq\left(\pi_{2}-\pi_{1}\right)}\int_{p\pi_{1}+\left(1-p\right)\pi_{2}}^{\pi_{2}}\Pi \left(x\right){\;}^{\pi_{2}}d_{p,q}x-\frac{1}{q}\Pi \left(\pi_{1}\right)\right]. \end{array}$$

By using (26)–(28) in (25), we obtain the desired identity (23). Thus, the proof is ended.

**Remark** **6.** *If we address p=1 in Lemma 2, then Lemma 2 reduces to ([15] Lemma 2).*

**Remark** **7.** *If we use p=1 and $q\to 1^{-}$ in Lemma 2, then Lemma 2 reduces to [40] (Lemma 2.1).*

**Theorem** **8.** *Suppose that the assumptions of Lemma 2 hold. If $\left|{\;}^{\pi_{2}}D_{p,q}\Pi \right|$ is a convex function over $\left[\pi_{1},\pi_{2}\right]$, then we have the following new inequality:*

(29) $$\begin{array}{ccc} & & \left|\int_{\pi_{1}p+\left(1-p\right)\pi_{2}}^{\pi_{2}}\Pi \left(x\right){\;}^{\pi_{2}}d_{p,q}x-\Pi \left(\frac{p\pi_{1}+q\pi_{2}}{{\left[2\right]}_{p,q}}\right)\right|\\ & \leq & \left(\pi_{2}-\pi_{1}\right)\left[\left(\left|{}^{\pi_{2}}D_{p,q}\Pi \left(\pi_{1}\right)\right|A_{1}\left(p,q\right)+\left|{}^{\pi_{2}}D_{p,q}\Pi \left(\pi_{2}\right)\right|A_{2}\left(p,q\right)\right)\right.\\ & & \left.+\left(\left|{}^{\pi_{2}}D_{p,q}\Pi \left(\pi_{1}\right)\right|A_{3}\left(p,q\right)+\left|{}^{\pi_{2}}D_{p,q}\Pi \left(\pi_{2}\right)\right|A_{4}\left(p,q\right)\right)\right] \end{array}$$

*where*

$$\begin{array}{ccc} A_{1}\left(p,q\right) & = & \frac{qp^{3}}{{\left({\left[2\right]}_{p,q}\right)}^{3}{\left[3\right]}_{p,q}},\\ A_{2}\left(p,q\right) & = & \frac{q\left(p^{3}\left(p^{2}+q^{2}-p\right)+p^{2}{\left[3\right]}_{p,q}\right)}{{\left({\left[2\right]}_{p,q}\right)}^{4}{\left[3\right]}_{p,q}},\\ A_{3}\left(p,q\right) & = & \frac{q\left(q+2p\right)}{{\left[2\right]}_{p,q}}-\frac{q^{2}\left(q^{2}+3p^{2}+3pq\right)}{{\left({\left[2\right]}_{p,q}\right)}^{3}{\left[3\right]}_{p,q}},\\ A_{4}\left(p,q\right) & = & \frac{q}{{\left[2\right]}_{p,q}}-\frac{q^{2}\left(q+2p\right)}{{\left({\left[2\right]}_{p,q}\right)}^{4}}-A_{3}\left(p,q\right). \end{array}$$

**Proof.**  Taking the modulus in Lemma 2 and using the convexity of $\left|{\;}^{\pi_{2}}D_{p,q}\Pi \right|$, we obtain that

(30) $$\begin{array}{ccc} & & \left|\int_{\pi_{1}p+\left(1-p\right)\pi_{2}}^{\pi_{2}}\Pi \left(x\right){\;}^{\pi_{2}}d_{p,q}x-\Pi \left(\frac{p\pi_{1}+q\pi_{2}}{{\left[2\right]}_{p,q}}\right)\right|\\ & \leq & \left(\pi_{2}-\pi_{1}\right)\left[\int_{0}^{\frac{p}{{\left[2\right]}_{p,q}}}qt\;\left|{}^{\pi_{2}}D_{p,q}\Pi \left(t\pi_{1}+\left(1-t\right)\pi_{2}\right)\right|d_{p,q}t\right.\\ & & \left.+\int_{\frac{p}{{\left[2\right]}_{p,q}}}^{1}\left(1-qt\right)\;\left|{}^{\pi_{2}}D_{p,q}\Pi \left(t\pi_{1}+\left(1-t\right)\pi_{2}\right)\right|d_{p,q}t\right]\\ & \leq & \left(\pi_{2}-\pi_{1}\right)\left[q\int_{0}^{\frac{p}{{\left[2\right]}_{p,q}}}t\left(t\left|{}^{\pi_{2}}D_{p,q}\Pi \left(\pi_{1}\right)\right|+\left(1-t\right)\left|{}^{\pi_{2}}D_{p,q}\Pi \left(\pi_{2}\right)\right|\right)d_{p,q}t\right.\\ & & \left.+\int_{\frac{p}{{\left[2\right]}_{p,q}}}^{1}\left(1-qt\right)\left(t\left|{}^{\pi_{2}}D_{p,q}\Pi \left(\pi_{1}\right)\right|+\left(1-t\right)\left|{}^{\pi_{2}}D_{p,q}\Pi \left(\pi_{2}\right)\right|\right)d_{p,q}t\right]. \end{array}$$

One can easily compute the integrals that appeared in the right side of the inequality (30)

(31) $$\begin{array}{ccc} \int_{0}^{\frac{p}{{\left[2\right]}_{p,q}}}t^{2}d_{p,q}t & = & \frac{p^{3}}{{\left({\left[2\right]}_{p,q}\right)}^{3}{\left[3\right]}_{p,q}}, \end{array}$$

(32) $$\begin{array}{ccc} \int_{0}^{\frac{p}{{\left[2\right]}_{p,q}}}t\left(1-t\right)d_{p,q}t & = & \frac{p^{3}\left(p^{2}+q^{2}-p\right)+p^{2}{\left[3\right]}_{p,q}}{{\left({\left[2\right]}_{p,q}\right)}^{4}{\left[3\right]}_{p,q}}, \end{array}$$

(33) $$\begin{array}{ccc} \int_{\frac{p}{{\left[2\right]}_{p,q}}}^{1}t\left(1-qt\right)d_{p,q}t & = & \frac{q\left(q+2p\right)}{{\left[2\right]}_{p,q}}-\frac{q^{2}\left(q^{2}+3p^{2}+3pq\right)}{{\left({\left[2\right]}_{p,q}\right)}^{3}{\left[3\right]}_{p,q}}, \end{array}$$

(34) $$\begin{array}{ccc} \int_{\frac{p}{{\left[2\right]}_{p,q}}}^{1}\left(1-t\right)\left(1-qt\right)d_{p,q}t & = & \frac{q}{{\left[2\right]}_{p,q}}-\frac{q^{2}\left(q+2p\right)}{{\left({\left[2\right]}_{p,q}\right)}^{3}}\\ & & -\left(\frac{q\left(q+2p\right)}{{\left[2\right]}_{p,q}}-\frac{q^{2}\left(q^{2}+3p^{2}+3pq\right)}{{\left({\left[2\right]}_{p,q}\right)}^{3}{\left[3\right]}_{p,q}}\right). \end{array}$$

Making use of (31)–(34) in (30) gives us the required inequality (29). Hence, the proof is finished. □

**Remark** **8.** *If we use p=1 in Theorem 8, then Theorem 8 becomes [15] (Theorem 5).*

**Remark** **9.** *If we take p=1 and $q\to 1^{-}$ in Theorem 8, then Theorem 8 reduces to [40] (Theorem 2.2)*

**Theorem** **9.** *Suppose that the assumptions of Lemma 2 hold. If ${\left|{\;}^{\pi_{2}}D_{p,q}\Pi \right|}^{r}$, r≥1 is a convex function over $\left[\pi_{1},\pi_{2}\right]$, then we have the following new inequality: *

(35) $$\begin{array}{ccc} & & \left|\int_{\pi_{1}p+\left(1-p\right)\pi_{2}}^{\pi_{2}}\Pi \left(x\right){\;}^{\pi_{2}}d_{p,q}x-\Pi \left(\frac{p\pi_{1}+q\pi_{2}}{{\left[2\right]}_{p,q}}\right)\right|\\ & \leq & \left(\pi_{2}-\pi_{1}\right){\left(\frac{p^{2}}{{\left({\left[2\right]}_{p,q}\right)}^{3}}\right)}^{1-\frac{1}{r}}\left[{\left\{{\left|{}^{\pi_{2}}D_{p,q}\Pi \left(\pi_{1}\right)\right|}^{r}A_{1}\left(p,q\right)+{\left|{}^{\pi_{2}}D_{p,q}\Pi \left(\pi_{2}\right)\right|}^{r}A_{2}\left(p,q\right)\right\}}^{\frac{1}{r}}\right.\\ & & \left.+{\left\{{\left|{}^{\pi_{2}}D_{p,q}\Pi \left(\pi_{1}\right)\right|}^{r}A_{3}\left(p,q\right)+{\left|{}^{\pi_{2}}D_{p,q}\Pi \left(\pi_{2}\right)\right|}^{r}A_{4}\left(p,q\right)\right\}}^{\frac{1}{r}}\right] \end{array}$$

*where $A_{1}\left(p,q\right)-A_{4}\left(p,q\right)$ are given in Theorem 8.*

**Proof.**  Taking the modulus in Lemma 2, applying the well-known power mean inequality for (p,q)-integrals, and by using the convexity of ${\left|{\;}^{\pi_{2}}D_{p,q}\Pi \right|}^{r}$, r≥1, we have

$$\begin{array}{ccc} & & \left|\int_{\pi_{1}p+\left(1-p\right)\pi_{2}}^{\pi_{2}}\Pi \left(x\right){\;}^{\pi_{2}}d_{p,q}x-\Pi \left(\frac{p\pi_{1}+q\pi_{2}}{{\left[2\right]}_{p,q}}\right)\right|\\ & \leq & \left(\pi_{2}-\pi_{1}\right)\left[\int_{0}^{\frac{p}{{\left[2\right]}_{p,q}}}qt\;\left|{}^{\pi_{2}}D_{p,q}\Pi \left(t\pi_{1}+\left(1-t\right)\pi_{2}\right)\right|d_{p,q}t\right.\\ & & \left.+\int_{\frac{p}{{\left[2\right]}_{p,q}}}^{1}\left(1-qt\right)\;\left|{}^{\pi_{2}}D_{p,q}\Pi \left(t\pi_{1}+\left(1-t\right)\pi_{2}\right)\right|d_{p,q}t\right]\\ & \leq & \left(\pi_{2}-\pi_{1}\right)\left[{\left(\int_{0}^{\frac{p}{{\left[2\right]}_{p,q}}}qtd_{p,q}t\right)}^{1-\frac{1}{r}}{\left\{q\int_{0}^{\frac{p}{{\left[2\right]}_{p,q}}}t\left(t{\left|{}^{\pi_{2}}D_{p,q}\Pi \left(\pi_{1}\right)\right|}^{r}+\left(1-t\right){\left|{\;}^{\pi_{2}}D_{p,q}\Pi \left(\pi_{2}\right)\right|}^{r}\right)d_{p,q}t\right\}}^{\frac{1}{r}}\right.\\ & & \left.+{\left(\int_{\frac{p}{{\left[2\right]}_{p,q}}}^{1}\left(1-qt\right)d_{p,q}t\right)}^{1-\frac{1}{r}}{\left\{q\int_{\frac{p}{{\left[2\right]}_{p,q}}}^{1}\left(1-qt\right)\left(t{\left|{}^{\pi_{2}}D_{p,q}\Pi \left(\pi_{1}\right)\right|}^{r}+\left(1-t\right){\left|{\;}^{\pi_{2}}D_{p,q}\Pi \left(\pi_{2}\right)\right|}^{r}\right)d_{p,q}t\right\}}^{\frac{1}{r}}\right]\\ & = & \left(\pi_{2}-\pi_{1}\right){\left(\frac{p^{2}}{{\left({\left[2\right]}_{p,q}\right)}^{3}}\right)}^{1-\frac{1}{r}}\left[{\left\{{\left|{}^{\pi_{2}}D_{p,q}\Pi \left(\pi_{1}\right)\right|}^{r}A_{1}\left(p,q\right)+{\left|{}^{\pi_{2}}D_{p,q}\Pi \left(\pi_{2}\right)\right|}^{r}A_{2}\left(p,q\right)\right\}}^{\frac{1}{r}}\right.\\ & & \left.+{\left\{{\left|{}^{\pi_{2}}D_{p,q}\Pi \left(\pi_{1}\right)\right|}^{r}A_{3}\left(p,q\right)+{\left|{}^{\pi_{2}}D_{p,q}\Pi \left(\pi_{2}\right)\right|}^{r}A_{4}\left(p,q\right)\right\}}^{\frac{1}{r}}\right] \end{array}$$

which ends the proof. □

**Remark** **10.** *If we put p=1 in Theorem 9, then Theorem 9 reduces to ([15] Theorem 6).*

**Remark** **11.** *If we set p=1 and $q\to 1^{-}$ in Theorem 9, then Theorem 9 becomes [18] (Corollary 2).*

**Theorem** **10.** *Suppose that the assumptions of Lemma 2 hold. If ${\left|{\;}^{\pi_{2}}D_{p,q}\Pi \right|}^{r}$, r>1 is a convex function over $\left[\pi_{1},\pi_{2}\right]$, then we have the following new inequality: *

(36) $$\begin{array}{ccc} & & \left|\int_{\pi_{1}p+\left(1-p\right)\pi_{2}}^{\pi_{2}}\Pi \left(x\right){\;}^{\pi_{2}}d_{p,q}x-\Pi \left(\frac{p\pi_{1}+q\pi_{2}}{{\left[2\right]}_{p,q}}\right)\right|\\ & \leq & q\left(\pi_{2}-\pi_{1}\right)\left[{\left({\left(\frac{p}{{\left[2\right]}_{p,q}}\right)}^{s+1}\left(\frac{p-q}{p^{s+1}-q^{s+1}}\right)\right)}^{\frac{1}{s}}{\left\{\begin{array}{c} {\left|{}^{\pi_{2}}D_{p,q}\Pi \left(\pi_{1}\right)\right|}^{r}\left(\frac{p^{2}}{{\left[2\right]}_{p,q}^{3}}\right)\\ +{\left|{}^{\pi_{2}}D_{p,q}\Pi \left(\pi_{2}\right)\right|}^{r}\left(\frac{p^{3}+pq^{2}+2p^{2}q-p^{2}}{{\left[2\right]}_{p,q}^{3}}\right) \end{array}\right\}}^{\frac{1}{r}}\right.\\ & & \left.+{\left(\int_{\frac{p}{{\left[2\right]}_{p,q}}}^{1}{\left(\frac{1}{q}-t\right)}^{s}d_{p,q}t\right)}^{\frac{1}{s}}{\left\{\begin{array}{c} {\left|{}^{\pi_{2}}D_{p,q}\Pi \left(\pi_{1}\right)\right|}^{r}\left(\frac{{\left[2\right]}_{p,q}-p^{2}}{{\left[2\right]}_{p,q}^{3}}\right)\\ +{\left|{}^{\pi_{2}}D_{p,q}\Pi \left(\pi_{2}\right)\right|}^{r}\left(\frac{q{\left[2\right]}_{p,q}^{2}+p^{2}-p-q}{{\left[2\right]}_{p,q}^{3}}\right) \end{array}\right\}}^{\frac{1}{r}}\right] \end{array}$$

*where s+r=sr.*

**Proof.**  Taking the modulus in Lemma 2, by applying the well-known Hölder’s inequality for definite (p,q) integrals and using the convexity of ${\left|{\;}^{\pi_{2}}D_{p,q}\Pi \right|}^{r}$, r>1, we obtain that

(37) $$\begin{array}{ccc} & & \left|\int_{\pi_{1}p+\left(1-p\right)\pi_{2}}^{\pi_{2}}\Pi \left(x\right){\;}^{\pi_{2}}d_{p,q}x-\Pi \left(\frac{p\pi_{1}+q\pi_{2}}{{\left[2\right]}_{p,q}}\right)\right|\\ & \leq & q\left(\pi_{2}-\pi_{1}\right)\left[\int_{0}^{\frac{p}{{\left[2\right]}_{p,q}}}t\;\left|{}^{\pi_{2}}D_{p,q}\Pi \left(t\pi_{1}+\left(1-t\right)\pi_{2}\right)\right|d_{p,q}t\right.\\ & & \left.+\int_{\frac{p}{{\left[2\right]}_{p,q}}}^{1}\left(\frac{1}{q}-t\right)\;\left|{}^{\pi_{2}}D_{p,q}\Pi \left(t\pi_{1}+\left(1-t\right)\pi_{2}\right)\right|d_{p,q}t\right]\\ & \leq & q\left(\pi_{2}-\pi_{1}\right)\left[{\left(\int_{0}^{\frac{p}{{\left[2\right]}_{p,q}}}t^{s}d_{p,q}t\right)}^{\frac{1}{s}}{\left\{\int_{0}^{\frac{p}{{\left[2\right]}_{p,q}}}\left(t{\left|{}^{\pi_{2}}D_{p,q}\Pi \left(\pi_{1}\right)\right|}^{r}+\left(1-t\right){\left|{\;}^{\pi_{2}}D_{p,q}\Pi \left(\pi_{2}\right)\right|}^{r}\right)d_{p,q}t\right\}}^{\frac{1}{r}}\right.\\ & & \left.+{\left(\int_{\frac{p}{{\left[2\right]}_{p,q}}}^{1}{\left(\frac{1}{q}-t\right)}^{s}d_{p,q}t\right)}^{\frac{1}{s}}{\left\{\int_{\frac{p}{{\left[2\right]}_{p,q}}}^{1}\left(t{\left|{}^{\pi_{2}}D_{p,q}\Pi \left(\pi_{1}\right)\right|}^{r}+\left(1-t\right){\left|{\;}^{\pi_{2}}D_{p,q}\Pi \left(\pi_{2}\right)\right|}^{r}\right)d_{p,q}t\right\}}^{\frac{1}{r}}\right]. \end{array}$$

One can easily evaluate the integrals that appear in the right side of the inequality (37)

(38) $$\begin{array}{ccc} {\left(\int_{0}^{\frac{p}{{\left[2\right]}_{p,q}}}t^{s}d_{p,q}t\right)}^{\frac{1}{s}} & = & {\left({\left(\frac{p}{{\left[2\right]}_{p,q}}\right)}^{s+1}\left(\frac{p-q}{p^{s+1}-q^{s+1}}\right)\right)}^{\frac{1}{s}} \end{array}$$

(39) $$\begin{array}{ccc} \int_{0}^{\frac{p}{{\left[2\right]}_{p,q}}}td_{p,q}t & = & \frac{p^{2}}{{\left[2\right]}_{p,q}^{3}}, \end{array}$$

(40) $$\begin{array}{ccc} \int_{0}^{\frac{p}{{\left[2\right]}_{p,q}}}\left(1-t\right)d_{p,q}t & = & \frac{p^{3}+pq^{2}+2p^{2}q-p^{2}}{{\left[2\right]}_{p,q}^{3}}, \end{array}$$

(41) $$\begin{array}{ccc} \int_{\frac{p}{{\left[2\right]}_{p,q}}}^{1}td_{p,q}t & = & \frac{{\left[2\right]}_{p,q}-p^{2}}{{\left[2\right]}_{p,q}^{3}}, \end{array}$$

(42) $$\begin{array}{ccc} \int_{\frac{p}{{\left[2\right]}_{p,q}}}^{1}\left(1-t\right)d_{p,q}t & = & \frac{q{\left[2\right]}_{p,q}^{2}+p^{2}-p-q}{{\left[2\right]}_{p,q}^{3}}. \end{array}$$

Making use of (38)–(42) gives us the required inequality (36). Hence, the proof is accomplished. □

**Corollary** **2.** *If we pick p=1 in Theorem 10, then we have the following new inequality*

$$\begin{array}{ccc} & & \left|\int_{\pi_{1}}^{\pi_{2}}\Pi \left(x\right){\;}^{\pi_{2}}d_{q}x-\Pi \left(\frac{\pi_{1}+q\pi_{2}}{{\left[2\right]}_{q}}\right)\right|\\ & \leq & q\left(\pi_{2}-\pi_{1}\right)\left[{\left({\left(\frac{1}{{\left[2\right]}_{q}}\right)}^{s+1}\left(\frac{1-q}{1-q^{s+1}}\right)\right)}^{\frac{1}{s}}{\left\{\begin{array}{c} {\left|{}^{\pi_{2}}D_{q}\Pi \left(\pi_{1}\right)\right|}^{r}\left(\frac{1}{{\left[2\right]}_{q}^{3}}\right)\\ +{\left|{}^{\pi_{2}}D_{q}\Pi \left(\pi_{2}\right)\right|}^{r}\left(\frac{q^{2}+2q}{{\left[2\right]}_{q}^{3}}\right) \end{array}\right\}}^{\frac{1}{r}}\right.\\ & & \left.+{\left(\int_{\frac{1}{{\left[2\right]}_{q}}}^{1}{\left(\frac{1}{q}-t\right)}^{s}d_{q}t\right)}^{\frac{1}{s}}{\left\{\begin{array}{c} {\left|{}^{\pi_{2}}D_{q}\Pi \left(\pi_{1}\right)\right|}^{r}\left(\frac{q}{{\left[2\right]}_{q}^{3}}\right)\\ +{\left|{}^{\pi_{2}}D_{q}\Pi \left(\pi_{2}\right)\right|}^{r}\left(\frac{q{\left[2\right]}_{q}^{2}-q}{{\left[2\right]}_{q}^{3}}\right) \end{array}\right\}}^{\frac{1}{r}}\right]. \end{array}$$

**Remark** **12.** *If we choose p=1 and $q\to 1^{-}$ in Theorem 10, then Theorem 10 reduces to [40] (Theorem 2.3).*

## 6. Trapezoidal-Type Inequalities through (p,q)${}^{\pi_{2}}$-Integral

In this section, we give some new trapezoidal inequalities by using the $\left(p,q\right)$ derivative and integral.

To prove the main results of this section, we need the following crucial lemma.

**Lemma** **3.** *Let $\Pi :\left[\pi_{1},\pi_{2}\right]\to R$ be a differentiable function on $\left(\pi_{1},\pi_{2}\right)$. If ${}^{\pi_{2}}D_{p,q}\Pi$ is continuous and integrable on $\left[\pi_{1},\pi_{2}\right]$, then we have the following identity:*

(43) $$\begin{array}{ccc} & & \frac{p\Pi \left(\pi_{1}\right)+q\Pi \left(\pi_{2}\right)}{{\left[2\right]}_{p,q}}-\frac{1}{p\left(\pi_{2}-\pi_{1}\right)}\int_{p\pi_{1}+\left(1-p\right)\pi_{2}}^{\pi_{2}}\Pi \left(x\right){\;}^{\pi_{2}}d_{p,q}x\\ & = & \frac{q\left(\pi_{2}-\pi_{1}\right)}{{\left[2\right]}_{p,q}}\int_{0}^{1}\left(1-{\left[2\right]}_{p,q}t\right){\;}^{\pi_{2}}D_{p,q}\Pi \left(t\pi_{1}+\left(1-t\right)\pi_{2}\right)\;d_{p,q}t \end{array}$$

*where 0<q<p≤1.*

**Proof.**  From (24) and the right side of (43), we obtain that

$$\begin{array}{ccc} & & \frac{q\left(\pi_{2}-\pi_{1}\right)}{{\left[2\right]}_{p,q}}\int_{0}^{1}\left(1-{\left[2\right]}_{p,q}t\right){\;}^{\pi_{2}}D_{p,q}\Pi \left(t\pi_{1}+\left(1-t\right)\pi_{2}\right)\;d_{p,q}t\\ & = & \frac{q\left(\pi_{2}-\pi_{1}\right)}{{\left[2\right]}_{p,q}}\left[\frac{1}{\left(\pi_{2}-\pi_{1}\right)\left(p-q\right)}\int_{0}^{1}\frac{\Pi \left(qt\pi_{1}+\left(1-qt\right)\pi_{2}\right)-\Pi \left(pt\pi_{1}+\left(1-pt\right)\pi_{2}\right)}{t}\;d_{p,q}t\right.\\ & & \left.-\frac{{\left[2\right]}_{p,q}}{\left(\pi_{2}-\pi_{1}\right)\left(p-q\right)}\int_{0}^{1}\left[\Pi \left(qt\pi_{1}+\left(1-qt\right)\pi_{2}\right)-\Pi \left(pt\pi_{1}+\left(1-pt\right)\pi_{2}\right)\right]\;d_{p,q}t\right]. \end{array}$$

From (27) and (28), we have

$$\begin{array}{ccc} & & \frac{q\left(\pi_{2}-\pi_{1}\right)}{{\left[2\right]}_{p,q}}\int_{0}^{1}\left(1-{\left[2\right]}_{p,q}t\right){\;}^{\pi_{2}}D_{p,q}\Pi \left(t\pi_{1}+\left(1-t\right)\pi_{2}\right)\;d_{p,q}t\\ & = & \frac{q\left(\pi_{2}-\pi_{1}\right)}{{\left[2\right]}_{p,q}}\left[\frac{\Pi \left(\pi_{2}\right)-\Pi \left(\pi_{1}\right)}{\pi_{2}-\pi_{1}}-\frac{{\left[2\right]}_{p,q}}{\pi_{2}-\pi_{1}}\left\{\frac{1}{pq\left(\pi_{2}-\pi_{1}\right)}\int_{p\pi_{1}+\left(1-p\right)\pi_{2}}^{\pi_{2}}\Pi \left(x\right){\;}^{\pi_{2}}d_{p,q}x-\frac{1}{q}\Pi \left(\pi_{1}\right)\right\}\right] \end{array}$$

where the identity (43) is obtained and the proof is accomplished. □

**Remark** **13.** *If we consider p=1 in Lemma 3, then Lemma 3 becomes [15] (Lemma 1).*

**Remark** **14.** *If we adopt p=1 and $q\to 1^{-}$ in Lemma 3, then Lemma 3 reduces to [41] (Lemma 2.1).*

**Theorem** **11.** *Suppose that the assumptions of Lemma 3 hold. If $\left|{\;}^{\pi_{2}}D_{p,q}\Pi \right|$ is a convex function over $\left[\pi_{1},\pi_{2}\right]$, then we have the following new inequality:*

(44) $$\begin{array}{ccc} & & \left|\frac{p\Pi \left(\pi_{1}\right)+q\Pi \left(\pi_{2}\right)}{{\left[2\right]}_{p,q}}-\frac{1}{p\left(\pi_{2}-\pi_{1}\right)}\int_{p\pi_{1}+\left(1-p\right)\pi_{2}}^{\pi_{2}}\Pi \left(x\right){\;}^{\pi_{2}}d_{p,q}x\right|\\ & \leq & \frac{q\left(\pi_{2}-\pi_{1}\right)}{{\left[2\right]}_{p,q}}\left[\left|{\;}^{\pi_{2}}D_{p,q}\Pi \left(\pi_{1}\right)\right|A_{5}\left(p,q\right)+\left|{\;}^{\pi_{2}}D_{p,q}\Pi \left(\pi_{2}\right)\right|A_{6}\left(p,q\right)\right] \end{array}$$

*where*

$$\begin{array}{ccc} A_{5}\left(p,q\right) & = & \int_{0}^{1}t\left|\left(1-{\left[2\right]}_{p,q}t\right)\right|\;d_{p,q}t,\\ A_{6}\left(p,q\right) & = & \int_{0}^{1}\left(1-t\right)\left|\left(1-{\left[2\right]}_{p,q}t\right)\right|\;d_{p,q}t. \end{array}$$

**Proof.**  Taking the modulus in Lemma 3 and using the convexity of $\left|{\;}^{\pi_{2}}D_{p,q}\Pi \right|,$ we have

(45) $$\begin{array}{ccc} & & \left|\frac{p\Pi \left(\pi_{1}\right)+q\Pi \left(\pi_{2}\right)}{{\left[2\right]}_{p,q}}-\frac{1}{p\left(\pi_{2}-\pi_{1}\right)}\int_{p\pi_{1}+\left(1-p\right)\pi_{2}}^{\pi_{2}}\Pi \left(x\right){\;}^{\pi_{2}}d_{p,q}x\right|\\ & \leq & \frac{q\left(\pi_{2}-\pi_{1}\right)}{{\left[2\right]}_{p,q}}\int_{0}^{1}t\left|\left(1-{\left[2\right]}_{p,q}t\right)\right|\;\left|{}^{\pi_{2}}D_{p,q}\Pi \left(\pi_{1}\right)\right|\;d_{p,q}t\\ & & +\int_{0}^{1}\left(1-t\right)\left|\left(1-{\left[2\right]}_{p,q}t\right)\right|\;\left|{}^{\pi_{2}}D_{p,q}\Pi \left(\pi_{2}\right)\right|\;d_{p,q}t\\ & = & \frac{q\left(\pi_{2}-\pi_{1}\right)}{{\left[2\right]}_{p,q}}\left[\left|{\;}^{\pi_{2}}D_{p,q}\Pi \left(\pi_{1}\right)\right|A_{5}\left(p,q\right)+\left|{\;}^{\pi_{2}}D_{p,q}\Pi \left(\pi_{2}\right)\right|A_{6}\left(p,q\right)\right] \end{array}$$

Thus, the proof is completed. □

**Remark** **15.** *If we set p=1 in Theorem 11, then Theorem 11 becomes [15] (Theorem 3).*

**Remark** **16.** *If we consider p=1 and $q\to 1^{-}$ in Theorem 11, then Theorem 11 reduces to [41] (Theorem 2.2).*

**Theorem** **12.** *Suppose that the assumptions of Lemma 3 hold. If ${\left|{\;}^{\pi_{2}}D_{p,q}\Pi \right|}^{r}$, r≥1 is a convex function over $\left[\pi_{1},\pi_{2}\right]$, then we have the following new inequality: *

(46) $$\begin{array}{ccc} & & \left|\frac{p\Pi \left(\pi_{1}\right)+q\Pi \left(\pi_{2}\right)}{{\left[2\right]}_{p,q}}-\frac{1}{p\left(\pi_{2}-\pi_{1}\right)}\int_{p\pi_{1}+\left(1-p\right)\pi_{2}}^{\pi_{2}}\Pi \left(x\right){\;}^{\pi_{2}}d_{p,q}x\right|\\ & \leq & \frac{q\left(\pi_{2}-\pi_{1}\right)}{{\left[2\right]}_{p,q}}{\left(\int_{0}^{1}\left|1-{\left[2\right]}_{p,q}t\right|\;d_{p,q}t\right)}^{1-\frac{1}{r}}{\left[{\left|{\;}^{\pi_{2}}D_{p,q}\Pi \left(\pi_{1}\right)\right|}^{r}A_{5}\left(p,q\right)+{\left|{\;}^{\pi_{2}}D_{p,q}\Pi \left(\pi_{2}\right)\right|}^{r}A_{6}\left(p,q\right)\right]}^{\frac{1}{r}} \end{array}$$

*where $A_{5}\left(p,q\right)$ and $A_{6}\left(p,q\right)$ are given in Theorem 11.*

**Proof.**  Taking the modulus in Lemma 3 and applying the well-known power mean inequality for (p,q) integrals and the convexity of ${\left|{\;}^{\pi_{2}}D_{p,q}\Pi \right|}^{r}$, r≥1, we get that.

(47) $$\begin{array}{ccc} & & \left|\frac{p\Pi \left(\pi_{1}\right)+q\Pi \left(\pi_{2}\right)}{{\left[2\right]}_{p,q}}-\frac{1}{p\left(\pi_{2}-\pi_{1}\right)}\int_{p\pi_{1}+\left(1-p\right)\pi_{2}}^{\pi_{2}}\Pi \left(x\right){\;}^{\pi_{2}}d_{p,q}x\right|\\ & \leq & \frac{q\left(\pi_{2}-\pi_{1}\right)}{{\left[2\right]}_{p,q}}{\left(\int_{0}^{1}\left|1-{\left[2\right]}_{p,q}t\right|\;d_{p,q}t\right)}^{1-\frac{1}{r}}{\left[\int_{0}^{1}\left|1-{\left[2\right]}_{p,q}t\right|\;{\left|{}^{\pi_{2}}D_{p,q}\Pi \left(t\pi_{1}+\left(1-t\right)\pi_{2}\right)\right|}^{r}\;d_{p,q}t\right]}^{\frac{1}{r}}\\ & \leq & \frac{q\left(\pi_{2}-\pi_{1}\right)}{{\left[2\right]}_{p,q}}{\left(\int_{0}^{1}\left|1-{\left[2\right]}_{p,q}t\right|\;d_{p,q}t\right)}^{1-\frac{1}{r}}\\ & & \times {\left[\int_{0}^{1}t\left|\left(1-{\left[2\right]}_{p,q}t\right)\right|\;{\left|{}^{\pi_{2}}D_{p,q}\Pi \left(\pi_{1}\right)\right|}^{r}\;d_{p,q}t+\int_{0}^{1}\left(1-t\right)\left|\left(1-{\left[2\right]}_{p,q}t\right)\right|\;{\left|{}^{\pi_{2}}D_{p,q}\Pi \left(\pi_{2}\right)\right|}^{r}\;d_{p,q}t\right]}^{\frac{1}{r}}\\ & = & \frac{q\left(\pi_{2}-\pi_{1}\right)}{{\left[2\right]}_{p,q}}{\left(\int_{0}^{1}\left|1-{\left[2\right]}_{p,q}t\right|\;d_{p,q}t\right)}^{1-\frac{1}{r}}{\left[{\left|{\;}^{\pi_{2}}D_{p,q}\Pi \left(\pi_{1}\right)\right|}^{r}A_{5}\left(p,q\right)+{\left|{\;}^{\pi_{2}}D_{p,q}\Pi \left(\pi_{2}\right)\right|}^{r}A_{6}\left(p,q\right)\right]}^{\frac{1}{r}}. \end{array}$$

Thus, the proof is finished. □

**Remark** **17.** *If we consider p=1 in Theorem 12, then Theorem 12 reduces to [15] (Theorem 4).*

**Remark** **18.** *If we address p=1 and $q\to 1^{-}$ in Theorem 12, then Theorem 12 becomes [42] (Theorem 1).*

**Theorem** **13.** *Suppose that the assumptions of Lemma 3 hold. If ${\left|{\;}^{\pi_{2}}D_{p,q}\Pi \right|}^{r}$, r>1 is a convex function over $\left[\pi_{1},\pi_{2}\right]$, then we have the following new inequality: *

(48) $$\begin{array}{ccc} & & \left|\frac{p\Pi \left(\pi_{1}\right)+q\Pi \left(\pi_{2}\right)}{{\left[2\right]}_{p,q}}-\frac{1}{p\left(\pi_{2}-\pi_{1}\right)}\int_{p\pi_{1}+\left(1-p\right)\pi_{2}}^{\pi_{2}}\Pi \left(x\right){\;}^{\pi_{2}}d_{p,q}x\right|\\ & \leq & \frac{q\left(\pi_{2}-\pi_{1}\right)}{{\left[2\right]}_{p,q}}{\left(\int_{0}^{1}{\left|1-{\left[2\right]}_{p,q}t\right|}^{s}\;d_{p,q}t\right)}^{\frac{1}{s}}{\left[\frac{{\left|{\;}^{\pi_{2}}D_{p,q}\Pi \left(\pi_{1}\right)\right|}^{r}+\left({\left[2\right]}_{p,q}-1\right){\left|{\;}^{\pi_{2}}D_{p,q}\Pi \left(\pi_{2}\right)\right|}^{r}}{{\left[2\right]}_{p,q}}\right]}^{\frac{1}{r}} \end{array}$$

*where s+r=sr.*

**Proof.**  Taking the modulus in Lemma 3 and applying the well-known Hölder’s inequality for (p,q)-integrals and the convexity of ${\left|{\;}^{\pi_{2}}D_{p,q}\Pi \right|}^{r}$, r>1, we obtain that

(49) $$\begin{array}{ccc} & & \left|\frac{p\Pi \left(\pi_{1}\right)+q\Pi \left(\pi_{2}\right)}{{\left[2\right]}_{p,q}}-\frac{1}{p\left(\pi_{2}-\pi_{1}\right)}\int_{p\pi_{1}+\left(1-p\right)\pi_{2}}^{\pi_{2}}\Pi \left(x\right){\;}^{\pi_{2}}d_{p,q}x\right|\\ & \leq & \frac{q\left(\pi_{2}-\pi_{1}\right)}{{\left[2\right]}_{p,q}}{\left(\int_{0}^{1}{\left|1-{\left[2\right]}_{p,q}t\right|}^{s}\;d_{p,q}t\right)}^{\frac{1}{s}}{\left[\int_{0}^{1}\;{\left|{}^{\pi_{2}}D_{p,q}\Pi \left(t\pi_{1}+\left(1-t\right)\pi_{2}\right)\right|}^{r}\;d_{p,q}t\right]}^{\frac{1}{r}}\\ & \leq & \frac{q\left(\pi_{2}-\pi_{1}\right)}{{\left[2\right]}_{p,q}}{\left(\int_{0}^{1}{\left|1-{\left[2\right]}_{p,q}t\right|}^{s}\;d_{p,q}t\right)}^{\frac{1}{s}}\\ & & \times {\left[\int_{0}^{1}t\;{\left|{}^{\pi_{2}}D_{p,q}\Pi \left(\pi_{1}\right)\right|}^{r}\;d_{p,q}t+\int_{0}^{1}\left(1-t\right)\;{\left|{}^{\pi_{2}}D_{p,q}\Pi \left(\pi_{2}\right)\right|}^{r}\;d_{p,q}t\right]}^{\frac{1}{r}}. \end{array}$$

We can calculate the integrals that occur in the right side of (49) as follows

(50) $$\begin{array}{ccc} \int_{0}^{1}t\;d_{p,q}t & = & \frac{1}{{\left[2\right]}_{p,q}}, \end{array}$$

(51) $$\begin{array}{ccc} \int_{0}^{1}\left(1-t\right)\;d_{p,q}t & = & \frac{{\left[2\right]}_{p,q}-1}{{\left[2\right]}_{p,q}}. \end{array}$$

Making use of (50) and (51) in (49) gives the desired result. Hence the proof is done. □

**Remark** **19.** *If we set p=1 and $q\to 1^{-}$ in Theorem 13, then Theorem 13 becomes [41] (Theorem 2.3).*

## 7. Applications to Special Means

For arbitrary positive numbers $\pi_{1},\pi_{2}$ ($\pi_{1}\neq \pi_{2}$), we consider the means as follows:

1. The arithmetic mean
  $$A=A\left(\pi_{1},\pi_{2}\right)=\frac{\pi_{1}+\pi_{2}}{2}.$$
2. The geometric mean
  $$G=G\left(\pi_{1},\pi_{2}\right)=\sqrt{\pi_{1}\pi_{2}}.$$
3. The harmonic means
  $$H=H\left(\pi_{1},\pi_{2}\right)=\frac{2\pi_{1}\pi_{2}}{\pi_{1}+\pi_{2}}.$$

**Proposition** **1.** *For $\pi_{1},\pi_{2}\in R$ with $\pi_{1}<\pi_{2}$ and 0<q<p≤1, the following inequality is true:*

$$A^{2}\left(\pi_{1},\pi_{2}\right)\leq A\left(\pi_{1}^{2},\pi_{2}^{2}\right)-p^{2}{\left(\pi_{2}-\pi_{1}\right)}^{2}\left[\frac{1}{{\left[2\right]}_{p,q}}-\frac{1}{{\left[3\right]}_{p,q}}\right]\leq A\left(\pi_{1}^{2},\pi_{2}^{2}\right).$$

**Proof.**  The inequality (17) for mapping $\Pi \left(x\right)=x^{2}$ leads to this conclusion. For verification, if we choose $\pi_{1}=0$, $\pi_{2}=1$, $p=\frac{3}{4}$, and $q=\frac{1}{2},$ we have

$$A^{2}\left(\pi_{1},\pi_{2}\right)=0.25,$$

$$A\left(\pi_{1}^{2},\pi_{2}^{2}\right)-p^{2}{\left(\pi_{2}-\pi_{1}\right)}^{2}\left[\frac{1}{{\left[2\right]}_{p,q}}-\frac{1}{{\left[3\right]}_{p,q}}\right]=0.4578,$$

and

$$A\left(\pi_{1}^{2},\pi_{2}^{2}\right)=0.5.$$

Thus,

0.25<0.4578<0.5

which shows that the inequality (17) is valid. □

**Proposition** **2.** *For $\pi_{1},\pi_{2}\in R$ with $\pi_{1}<\pi_{2}$ and 0<q<p≤1, the following inequality is true:*

$$G^{-2}\left(\pi_{1},\pi_{2}\right)H\left(\pi_{1},\pi_{2}\right)\leq A\left(\Theta_{1},\Theta_{2}\right)\leq H^{-1}\left(\pi_{1},\pi_{2}\right),$$

*where*

$$\Theta_{1}=\left(p-q\right)\sum_{n=0}^{\infty }\frac{q^{n}}{p^{n+1}}{\left(\frac{q^{n}}{p^{n+1}}\left(\pi_{1}+p\left(\pi_{2}-\pi_{1}\right)\right)+\left(1-\frac{q^{n}}{p^{n+1}}\right)\pi_{1}\right)}^{-1}$$

*and*

$$\Theta_{2}=\left(p-q\right)\sum_{n=0}^{\infty }\frac{q^{n}}{p^{n+1}}{\left(\frac{q^{n}}{p^{n+1}}\left(\pi_{2}+p\left(\pi_{1}-\pi_{2}\right)\right)+\left(1-\frac{q^{n}}{p^{n+1}}\right)\pi_{2}\right)}^{-1}.$$

**Proof.**  The inequality (17) for mapping $\Pi \left(x\right)=\frac{1}{x}$, where x≠0 leads to this conclusion. □

**Proposition** **3.** *For $\pi_{1},\pi_{2}\in R$ with $\pi_{1}<\pi_{2}$ and 0<q<p≤1, the following inequality is true:*

$$\ln\left(A\left(\pi_{1},\pi_{2}\right)\right)\leq A\left(\Theta_{3},\Theta_{4}\right)\leq \ln G\left(\pi_{1},\pi_{2}\right),$$

*where*

$$\Theta_{3}=\left(p-q\right)\sum_{n=0}^{\infty }\frac{q^{n}}{p^{n+1}}\ln\left(\frac{q^{n}}{p^{n+1}}\left(\pi_{1}+p\left(\pi_{2}-\pi_{1}\right)\right)+\left(1-\frac{q^{n}}{p^{n+1}}\right)\pi_{1}\right)$$

*and*

$$\Theta_{4}=\left(p-q\right)\sum_{n=0}^{\infty }\frac{q^{n}}{p^{n+1}}\ln\left(\frac{q^{n}}{p^{n+1}}\left(\pi_{2}+p\left(\pi_{1}-\pi_{2}\right)\right)+\left(1-\frac{q^{n}}{p^{n+1}}\right)\pi_{2}\right).$$

**Proof.**  The inequality (17) for mapping $\Pi \left(x\right)=\ln x$ leads to this conclusion. □

## 8. Conclusions

In the present research, we used the notions of $\left(p,q\right)$ derivative and integral, some new HH-type inequalities, and estimates for midpoint and trapezoidal type inequalities are derived. To approve their generalized behavior, we show the connection between our outcomes and the already established ones. Moreover, we provided applications to special means using the newly proved inequalities to show their significance. In future works, researchers can obtain comparable results by utilizing different kinds of convexity.

## Data Availability

No data were used to support this study.
